# Crystal structure of 2-di­methyl­amino-1-eth­oxy­carbonyl-3-methyl-3,4,5,6-tetra­hydro­pyrimidin-1-ium tetra­phenyl­borate

**DOI:** 10.1107/S2056989015020034

**Published:** 2015-10-31

**Authors:** Ioannis Tiritiris, Willi Kantlehner

**Affiliations:** a Fakultät Chemie/Organische Chemie, Hochschule Aalen, Beethovenstrasse 1, D-73430 Aalen, Germany

**Keywords:** crystal structure, cyclic guanidinium salt, tetra­phenyl­borate, C—H⋯π inter­actions

## Abstract

The asymmetric unit of the title salt, C_10_H_20_N_3_O_2_^+^·C_24_H_20_B^−^, contains two cations and two tetra­phenyl­borate ions. The C—N bond lengths in the central CN_3_ unit of the guanidinium ions range between 1.323 (2) and 1.381 (2) Å, indicating partial double-bond character. The central C atoms are bonded to the three N atoms in a nearly ideal trigonal–planar geometry and the positive charge is delocalized in the CN_3_ plane. The cationic six-membered rings are nonplanar, the dihedral angles between the N/C/N and C/C/C planes ranging from 45.8 (1) to 53.6 (1)°. In the crystal, C—H⋯π inter­actions are present between the guanidinium H atoms and the phenyl rings of the tetra­phenyl­borate ions. The phenyl rings form aromatic pockets, in which the guanidinium ions are embedded.

## Related literature   

For the crystal structures of alkali metal tetra­phenyl­borates, see: Behrens *et al.* (2012 ▸). For the crystal structure of 2-di­methyl­amino-1-(2-eth­oxy-2-oxoeth­yl)-3-methyl-3,4,5,6-tetra­hydro­pyrimidin-1-ium tetra­phenyl­borate, see: Tiritiris & Kantlehner (2012*a* ▸). For the crystal structure of 1-benzyl-2-di­methyl­amino-3-methyl-3,4,5,6-tetra­hydro­pyrimidin-1-ium bromide, see: Tiritiris & Kantlehner, 2012*b* ▸.
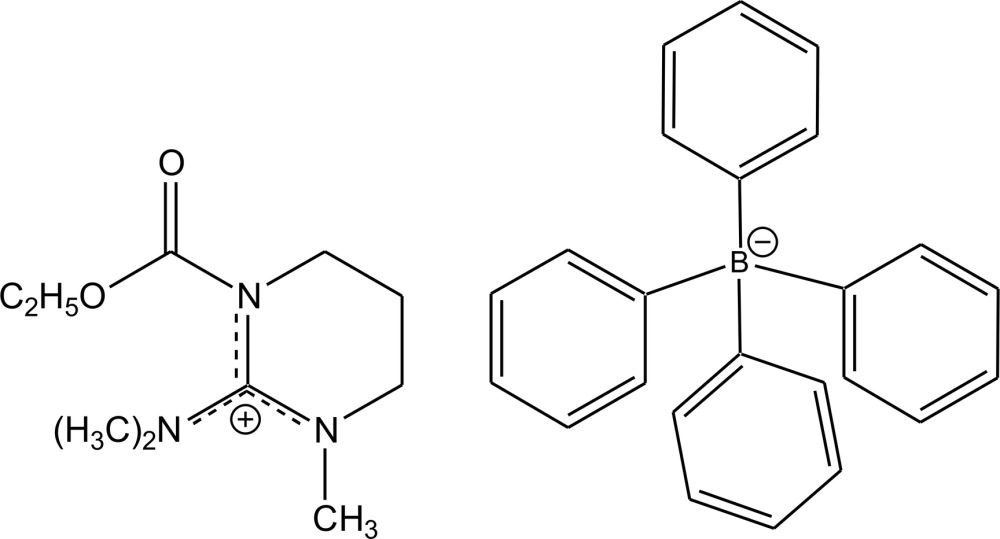


## Experimental   

### Crystal data  

C_10_H_20_N_3_O_2_^+^·C_24_H_20_B^−^
*M* *_r_* = 533.50
Monoclinic 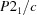

*a* = 18.5710 (5) Å
*b* = 19.2164 (7) Å
*c* = 18.6107 (5) Å
β = 119.280 (2)°
*V* = 5793.0 (3) Å^3^
*Z* = 8
Mo *K*α radiation
μ = 0.08 mm^−1^
*T* = 100 K
0.24 × 0.17 × 0.12 mm

### Data collection  

Bruker–Nonius KappaCCD diffractometer
26283 measured reflections
14001 independent reflections
9686 reflections with *I* > 2σ(*I*)
*R*_int_ = 0.058

### Refinement  

*R*[*F* ^2^ > 2σ(*F* ^2^)] = 0.059
*wR*(*F* ^2^) = 0.119
*S* = 1.04
14001 reflections
729 parameters
H-atom parameters constrained
Δρ_max_ = 0.50 e Å^−3^
Δρ_min_ = −0.25 e Å^−3^

### 

Data collection: *COLLECT* (Hooft, 2004 ▸); cell refinement: *SCALEPACK* (Otwinowski & Minor, 1997 ▸); data reduction: *SCALEPACK*; program(s) used to solve structure: *SHELXS97* (Sheldrick, 2008 ▸); program(s) used to refine structure: *SHELXL2014* (Sheldrick, 2015 ▸); molecular graphics: *DIAMOND* (Brandenburg & Putz, 2005 ▸); software used to prepare material for publication: *SHELXL2014*.

## Supplementary Material

Crystal structure: contains datablock(s) I, global. DOI: 10.1107/S2056989015020034/zl2648sup1.cif

Structure factors: contains datablock(s) I. DOI: 10.1107/S2056989015020034/zl2648Isup2.hkl

Supporting information file. DOI: 10.1107/S2056989015020034/zl2648Isup3.cml

**Fig. 1.**
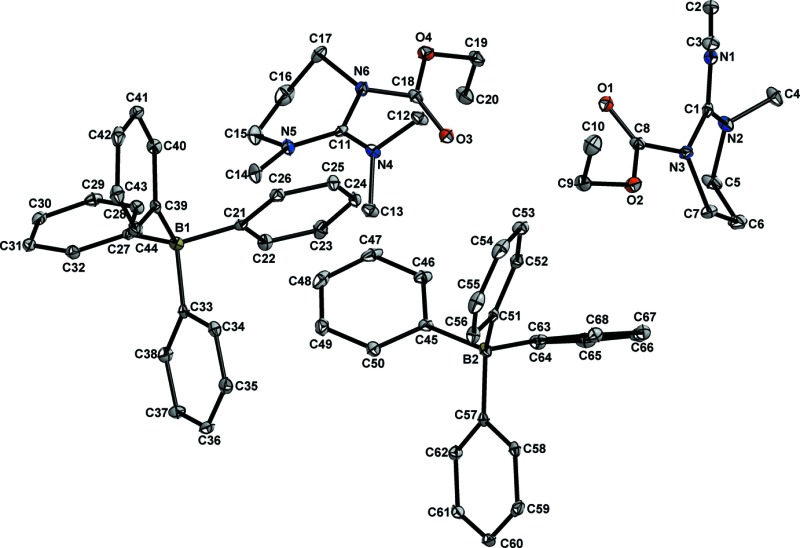
The structure of the title compound with displacement ellipsoids at the 50% probability level. All hydrogen atoms were omitted for the sake of clarity.

. DOI: 10.1107/S2056989015020034/zl2648fig1.tif
The structure of the title compound with displacement ellipsoids at the 50% probability level. All hydrogen atoms were omitted for the sake of clarity.

**Fig. 2.**
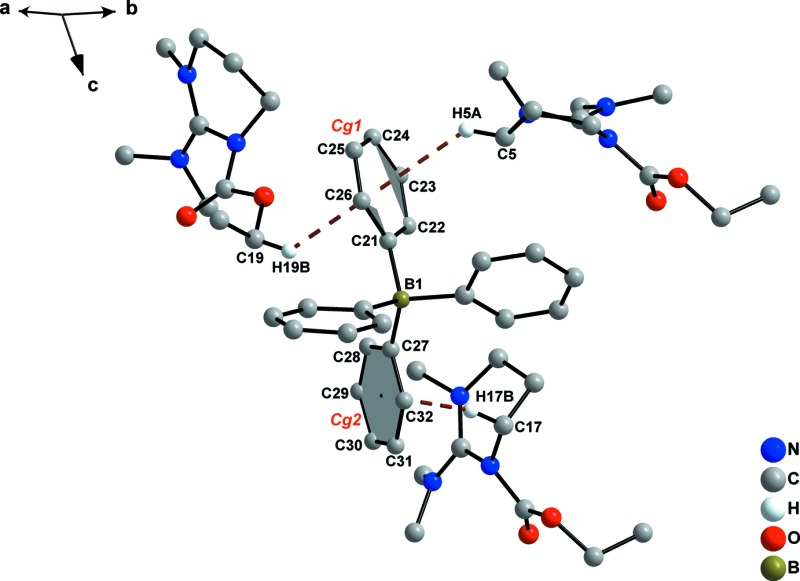
C—H···π interactions (brown dashed lines) between the hydrogen atoms of the guanidinium ion and the phenyl carbon atoms (centroids) of the tetraphenylborate ion (anion I).

. DOI: 10.1107/S2056989015020034/zl2648fig2.tif
C—H⋯π inter­actions (brown dashed lines) between the hydrogen atoms of the guanidinium ion and the phenyl carbon atoms (centroids) of the tetra­phenyl­borate ion (anion I).

**Fig. 3.**
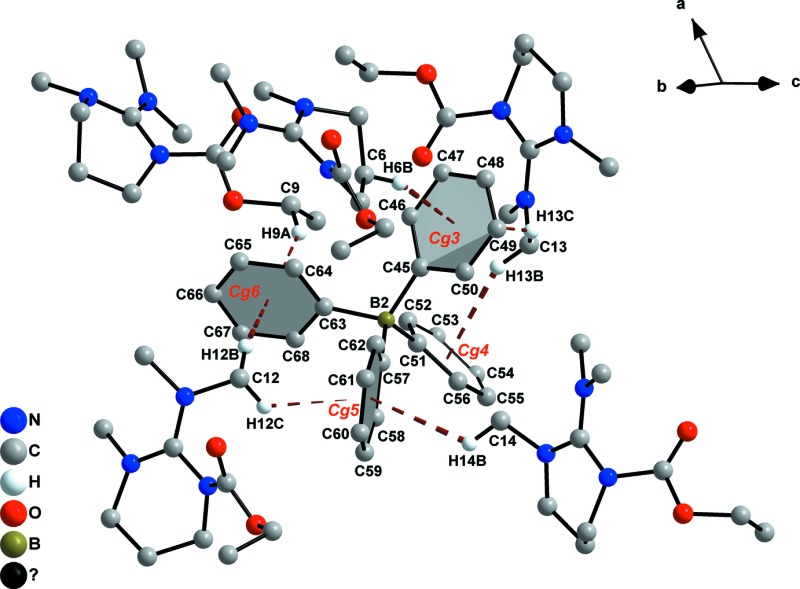
C—H···π interactions (brown dashed lines) between the hydrogen atoms of the guanidinium ion and the phenyl carbon atoms (centroids) of the tetraphenylborate ion (anion II).

. DOI: 10.1107/S2056989015020034/zl2648fig3.tif
C—H⋯π inter­actions (brown dashed lines) between the hydrogen atoms of the guanidinium ion and the phenyl carbon atoms (centroids) of the tetra­phenyl­borate ion (anion II).

CCDC reference: 1432704

Additional supporting information:  crystallographic information; 3D view; checkCIF report

## Figures and Tables

**Table 1:** Hydrogen-bond geometry (, ) *Cg*1, *Cg*2, *Cg*3, *Cg*4, *Cg*5 and *Cg*6 are the centroids of the C21C26, C27C32, C45C50, C51C56, C57C62 and C63C68 rings, respectively.

*D*H*A*	*D*H	H*A*	*D* *A*	*D*H*A*
C5H5*A* *Cg*1	0.99	2.77	3.502(3)	131
C19H19*B* *Cg*1	0.99	2.86	3.559(3)	128
C17H17*B* *Cg*2	0.99	2.67	3.632(3)	165
C6H6*B* *Cg*3	0.99	2.75	3.624(3)	147
C13H13*C* *Cg*3	0.98	2.72	3.421(3)	129
C13H13*B* *Cg*4	0.98	2.67	3.583(3)	154
C12H12*C* *Cg*5	0.98	2.70	3.329(3)	121
C14H14*B* *Cg*5	0.98	2.76	3.354(3)	120
C12H12*B* *Cg*6	0.98	2.64	3.535(3)	151
C9H9*A* *Cg*6	0.99	2.75	3.655(3)	151

## supplementary crystallographic information

### S1. Comment

The cation in the title compound is similar to the cations in the structurally known compounds 2-dimethylamino-1-ethoxycarbonyl-3-methyl-3,4,5,6-tetrahydropyrimidin-1-ium tetraphenylborate (Tiritiris & Kantlehner, 2012*a*) and 1-benzyl-2-dimethylamino-3-methyl-3,4,5,6-tetrahydropyrimidin-1-ium bromide (Tiritiris & Kantlehner, 2012*b*). The asymmetric unit contains two cations and two tetraphenylborate ions. Prominent bond parameters in the guanidinium ions are: C1–N1 = 1.323 (2) Å, C1–N2 = 1.333 (2) Å, C1–N3 = 1.379 (2) Å (cation I) and C11–N4 = 1.326 (2) Å, C11–N5 = 1.324 (2) Å, C11–N6 = 1.381 (2) Å (cation II). The N–C–N angles are in a range from 116.8 (2)° to 122.7 (2)°, indicating nearly ideal trigonal-planar surroundings of the carbon centres C1 and C11 by the nitrogen atoms. The positive charge is completely delocalized in the CN_3_ plane. The cyclic guanidinium ions are non planar (Fig. 1). The carbon atoms C6 and C16 are not in the ring plane. The dihedral angle between the planes N3/C1/N2 and C5/C6/C7 is 53.6 (1)°, the dihedral angle between the planes N5/C11/N6 and C15/C16/C17 is 45.8 (1)°. These values are comparable with those determined for the guanidinium ions in 2-dimethylamino-1-(2-ethoxy-2-oxoethyl)-3-methyl-3,4,5,6-tetrahydropyrimidin-1-ium tetraphenylborate [49.9 (1)°] (Tiritiris & Kantlehner, 2012*a*) and 1-benzyl-2-dimethylamino-3-methyl-3,4,5,6-tetrahydropyrimidin-1-ium bromide [55.0 (3)°] (Tiritiris & Kantlehner, 2012*b*). The bond lengths and angles in the tetraphenylborate ions are in good agreement with the data from the crystal structure analysis of the alkali metal tetraphenylborates (Behrens *et al.*, 2012). C–H···π interactions between the guanidinium hydrogen atoms of –N(CH_3_)_2_ and –CH_2_ groups and the phenyl carbon atoms (centroids) of the tetraphenylborate ion are present (Fig. 2 and 3), ranging from 2.64 to 2.86 Å (Tab. 1). The phenyl rings form aromatic pockets, in which the guanidinium ions are embedded.

### S2. Experimental

The title compound was obtained by reaction of 1-methyl-2-dimethylamino-1,4,5,6-tetrahydropyrimidine with chloroformic acid ethyl ester in acetonitrile for one hour at room temperature. After evaporation of the solvent the crude 2-dimethylamino-1-(2-ethoxy-2-oxomethyl)-3-methyl-3,4,5,6-tetrahydropyrimidin-1-ium chloride (I) was washed with diethylether and dried *in vacuo*. 1.0 g (4.0 mmol) of (I) was dissolved in 20 ml acetonitrile and 1.37 g (4.0 mmol) of sodium tetraphenylborate in 10 ml acetonitrile was added. After stirring for one hour at room temperature, the precipitated sodium chloride was filtered off. The title compound crystallized from a saturated acetonitrile solution after several days at 273 K, forming colorless single crystals. Yield: 1.67 g (77.5%).

### S3. Refinement

The hydrogen atoms of the methyl groups were allowed to rotate with a fixed angle around the C–N and C–C bonds to best fit the experimental electron density, with *U*_iso_(H) set to 1.5 *U*_eq_(C) and d(C—H) = 0.98 Å. The remaining H atoms were placed in calculated positions with d(C—H) = 0.99 Å (H atoms in CH_2_ groups) and (C—H) = 0.95 Å (H atoms in aromatic rings). They were refined using a riding model, with *U*_iso_(H) set to 1.2*U*_eq_(C).

### Crystal data

**Table:**

C_10_H_20_N_3_O_2_^+^·C_24_H_20_B^−^	*F*(000) = 2288
*M**_r_* = 533.50	*D*_x_ = 1.223 Mg m^−^^3^
Monoclinic, *P*2_1_/*c*	Mo *K*α radiation, λ = 0.71073 Å
Hall symbol: -P 2ybc	Cell parameters from 26101 reflections
*a* = 18.5710 (5) Å	θ = 1.3–28.1°
*b* = 19.2164 (7) Å	µ = 0.08 mm^−^^1^
*c* = 18.6107 (5) Å	*T* = 100 K
β = 119.280 (2)°	Block, colorless
*V* = 5793.0 (3) Å^3^	0.24 × 0.17 × 0.12 mm
*Z* = 8	

### Data collection

**Table:**

Bruker–Nonius KappaCCD diffractometer	9686 reflections with *I* > 2σ(*I*)
Radiation source: sealed tube	*R*_int_ = 0.058
Graphite monochromator	θ_max_ = 28.2°, θ_min_ = 1.3°
φ scans, and ω scans	*h* = −24→24
26283 measured reflections	*k* = −25→23
14001 independent reflections	*l* = −24→24

### Refinement

**Table:**

Refinement on *F*^2^	Primary atom site location: structure-invariant direct methods
Least-squares matrix: full	Secondary atom site location: difference Fourier map
*R*[*F*^2^ > 2σ(*F*^2^)] = 0.059	Hydrogen site location: inferred from neighbouring sites
*wR*(*F*^2^) = 0.119	H-atom parameters constrained
*S* = 1.03	*w* = 1/[σ^2^(*F*_o_^2^) + (0.0219*P*)^2^ + 5.1462*P*] where *P* = (*F*_o_^2^ + 2*F*_c_^2^)/3
14001 reflections	(Δ/σ)_max_ < 0.001
729 parameters	Δρ_max_ = 0.50 e Å^−^^3^
0 restraints	Δρ_min_ = −0.25 e Å^−^^3^

### Special details

**Table:**

Geometry. All e.s.d.'s (except the e.s.d. in the dihedral angle between two l.s. planes) are estimated using the full covariance matrix. The cell e.s.d.'s are taken into account individually in the estimation of e.s.d.'s in distances, angles and torsion angles; correlations between e.s.d.'s in cell parameters are only used when they are defined by crystal symmetry. An approximate (isotropic) treatment of cell e.s.d.'s is used for estimating e.s.d.'s involving l.s. planes.
Refinement. Refinement of *F*^2^ against ALL reflections. The weighted *R*-factor *wR* and goodness of fit *S* are based on *F*^2^, conventional *R*-factors *R* are based on *F*, with *F* set to zero for negative *F*^2^. The threshold expression of *F*^2^ > σ(*F*^2^) is used only for calculating *R*-factors(gt) *etc*. and is not relevant to the choice of reflections for refinement. *R*-factors based on *F*^2^ are statistically about twice as large as those based on *F*, and *R*-factors based on ALL data will be even larger.

### Fractional atomic coordinates and isotropic or equivalent isotropic displacement parameters (Å^2^)

**Table:**

	*x*	*y*	*z*	*U*_iso_*/*U*_eq_	
N1	0.79842 (9)	0.55416 (8)	−0.00502 (9)	0.0141 (3)	
N2	0.81433 (9)	0.67471 (8)	0.00009 (9)	0.0157 (3)	
N3	0.72182 (9)	0.62582 (8)	0.03487 (9)	0.0135 (3)	
C1	0.77986 (10)	0.61714 (9)	0.00972 (10)	0.0129 (3)	
C2	0.87832 (12)	0.53755 (11)	0.00129 (11)	0.0204 (4)	
H2A	0.8711	0.5301	−0.0540	0.031*	
H2B	0.9006	0.4952	0.0342	0.031*	
H2C	0.9167	0.5762	0.0279	0.031*	
C3	0.74394 (12)	0.49416 (9)	−0.02357 (12)	0.0195 (4)	
H3A	0.7667	0.4625	0.0238	0.029*	
H3B	0.7395	0.4697	−0.0718	0.029*	
H3C	0.6891	0.5099	−0.0351	0.029*	
C4	0.83819 (13)	0.68127 (11)	−0.06387 (12)	0.0214 (4)	
H4A	0.8189	0.6404	−0.0999	0.032*	
H4B	0.8984	0.6845	−0.0380	0.032*	
H4C	0.8132	0.7233	−0.0964	0.032*	
C5	0.79627 (12)	0.73980 (10)	0.03049 (12)	0.0207 (4)	
H5A	0.8229	0.7799	0.0193	0.025*	
H5B	0.8172	0.7369	0.0906	0.025*	
C6	0.70353 (12)	0.74823 (10)	−0.01491 (12)	0.0216 (4)	
H6A	0.6826	0.7484	−0.0751	0.026*	
H6B	0.6881	0.7929	0.0005	0.026*	
C7	0.66602 (11)	0.68779 (9)	0.00827 (11)	0.0156 (4)	
H7A	0.6558	0.7023	0.0536	0.019*	
H7B	0.6123	0.6751	−0.0397	0.019*	
C8	0.71983 (11)	0.58034 (9)	0.09302 (11)	0.0142 (3)	
O1	0.77527 (8)	0.54207 (7)	0.13625 (8)	0.0191 (3)	
O2	0.64801 (8)	0.58761 (7)	0.09186 (8)	0.0184 (3)	
C9	0.63793 (12)	0.54654 (10)	0.15269 (12)	0.0190 (4)	
H9A	0.5975	0.5697	0.1650	0.023*	
H9B	0.6913	0.5439	0.2045	0.023*	
C10	0.60852 (13)	0.47449 (11)	0.12107 (13)	0.0257 (4)	
H10A	0.5553	0.4771	0.0702	0.039*	
H10B	0.6020	0.4479	0.1625	0.039*	
H10C	0.6490	0.4513	0.1098	0.039*	
N4	0.63551 (9)	0.36665 (8)	0.40962 (9)	0.0135 (3)	
N5	0.73941 (10)	0.38338 (8)	0.54432 (9)	0.0182 (3)	
N6	0.77452 (9)	0.36757 (8)	0.44206 (9)	0.0150 (3)	
C11	0.71453 (11)	0.37415 (9)	0.46510 (10)	0.0124 (3)	
C12	0.60942 (11)	0.32737 (10)	0.33341 (11)	0.0170 (4)	
H12A	0.5936	0.3598	0.2875	0.025*	
H12B	0.5621	0.2980	0.3227	0.025*	
H12C	0.6551	0.2980	0.3391	0.025*	
C13	0.56893 (11)	0.40535 (10)	0.41317 (11)	0.0167 (4)	
H13A	0.5341	0.3729	0.4232	0.025*	
H13B	0.5354	0.4294	0.3607	0.025*	
H13C	0.5930	0.4395	0.4579	0.025*	
C14	0.68755 (14)	0.36456 (12)	0.58036 (12)	0.0276 (5)	
H14A	0.6620	0.4066	0.5878	0.041*	
H14B	0.7215	0.3422	0.6339	0.041*	
H14C	0.6444	0.3322	0.5436	0.041*	
C15	0.82339 (12)	0.40587 (12)	0.60501 (12)	0.0268 (5)	
H15A	0.8453	0.3740	0.6529	0.032*	
H15B	0.8204	0.4530	0.6248	0.032*	
C16	0.88247 (13)	0.40729 (11)	0.57137 (13)	0.0273 (5)	
H16A	0.8796	0.4529	0.5453	0.033*	
H16B	0.9397	0.4002	0.6164	0.033*	
C17	0.85854 (11)	0.35009 (10)	0.50887 (11)	0.0174 (4)	
H17A	0.8977	0.3477	0.4872	0.021*	
H17B	0.8584	0.3046	0.5338	0.021*	
C18	0.76607 (11)	0.40591 (9)	0.37379 (10)	0.0144 (3)	
O3	0.70768 (8)	0.44185 (7)	0.33087 (7)	0.0180 (3)	
O4	0.83231 (8)	0.39572 (7)	0.36536 (8)	0.0181 (3)	
C19	0.83792 (12)	0.43919 (10)	0.30418 (11)	0.0174 (4)	
H19A	0.7827	0.4434	0.2549	0.021*	
H19B	0.8754	0.4171	0.2872	0.021*	
C20	0.86987 (13)	0.51034 (10)	0.33867 (12)	0.0224 (4)	
H20A	0.8303	0.5338	0.3509	0.034*	
H20B	0.8771	0.5377	0.2982	0.034*	
H20C	0.9230	0.5059	0.3893	0.034*	
B1	0.98280 (12)	0.63490 (10)	0.78042 (11)	0.0113 (4)	
C21	0.98217 (10)	0.64626 (9)	0.69255 (10)	0.0125 (3)	
C22	1.01083 (11)	0.70816 (10)	0.67468 (11)	0.0158 (4)	
H22	1.0341	0.7430	0.7161	0.019*	
C23	1.00657 (11)	0.72050 (10)	0.59924 (11)	0.0193 (4)	
H23	1.0278	0.7626	0.5903	0.023*	
C24	0.97130 (11)	0.67145 (10)	0.53708 (10)	0.0176 (4)	
H24	0.9684	0.6794	0.4854	0.021*	
C25	0.94022 (11)	0.61059 (10)	0.55120 (11)	0.0178 (4)	
H25	0.9148	0.5770	0.5086	0.021*	
C26	0.94618 (11)	0.59863 (9)	0.62756 (10)	0.0145 (3)	
H26	0.9249	0.5563	0.6360	0.017*	
C27	1.07086 (10)	0.65452 (9)	0.86183 (10)	0.0122 (3)	
C28	1.14423 (11)	0.67309 (9)	0.86286 (10)	0.0147 (3)	
H28	1.1443	0.6760	0.8119	0.018*	
C29	1.21742 (11)	0.68755 (9)	0.93552 (11)	0.0169 (4)	
H29	1.2658	0.7000	0.9333	0.020*	
C30	1.21940 (11)	0.68370 (9)	1.01061 (11)	0.0163 (4)	
H30	1.2688	0.6937	1.0603	0.020*	
C31	1.14793 (11)	0.66496 (9)	1.01231 (11)	0.0170 (4)	
H31	1.1484	0.6617	1.0635	0.020*	
C32	1.07584 (11)	0.65097 (9)	0.93950 (10)	0.0149 (3)	
H32	1.0278	0.6385	0.9423	0.018*	
C33	0.90974 (10)	0.68769 (9)	0.77216 (10)	0.0114 (3)	
C34	0.82648 (11)	0.67327 (9)	0.71577 (10)	0.0141 (3)	
H34	0.8133	0.6307	0.6862	0.017*	
C35	0.76280 (11)	0.71868 (10)	0.70163 (11)	0.0169 (4)	
H35	0.7074	0.7065	0.6636	0.020*	
C36	0.77995 (11)	0.78203 (10)	0.74298 (11)	0.0186 (4)	
H36	0.7367	0.8134	0.7338	0.022*	
C37	0.86115 (12)	0.79827 (10)	0.79769 (11)	0.0198 (4)	
H37	0.8740	0.8417	0.8256	0.024*	
C38	0.92447 (11)	0.75190 (9)	0.81246 (11)	0.0156 (4)	
H38	0.9796	0.7643	0.8512	0.019*	
C39	0.96464 (10)	0.55282 (9)	0.79114 (10)	0.0125 (3)	
C40	1.01674 (11)	0.50062 (10)	0.78853 (11)	0.0168 (4)	
H40	1.0613	0.5141	0.7801	0.020*	
C41	1.00571 (12)	0.43049 (10)	0.79772 (11)	0.0197 (4)	
H41	1.0417	0.3970	0.7945	0.024*	
C42	0.94228 (13)	0.40910 (10)	0.81155 (11)	0.0220 (4)	
H42	0.9343	0.3611	0.8175	0.026*	
C43	0.89074 (12)	0.45828 (10)	0.81660 (11)	0.0194 (4)	
H43	0.8475	0.4442	0.8268	0.023*	
C44	0.90227 (11)	0.52917 (9)	0.80670 (11)	0.0161 (4)	
H44	0.8664	0.5623	0.8107	0.019*	
B2	0.46587 (12)	0.61087 (10)	0.28109 (11)	0.0103 (4)	
C45	0.54736 (10)	0.59612 (9)	0.37117 (10)	0.0128 (3)	
C46	0.62523 (11)	0.57874 (9)	0.38126 (11)	0.0172 (4)	
H46	0.6325	0.5783	0.3342	0.021*	
C47	0.69209 (11)	0.56207 (10)	0.45750 (12)	0.0220 (4)	
H47	0.7439	0.5509	0.4618	0.026*	
C48	0.68321 (12)	0.56176 (10)	0.52710 (12)	0.0243 (4)	
H48	0.7289	0.5509	0.5794	0.029*	
C49	0.60724 (13)	0.57734 (10)	0.51991 (11)	0.0225 (4)	
H49	0.6003	0.5766	0.5672	0.027*	
C50	0.54087 (11)	0.59417 (10)	0.44328 (11)	0.0174 (4)	
H50	0.4892	0.6048	0.4396	0.021*	
C51	0.42408 (10)	0.53399 (9)	0.24948 (10)	0.0118 (3)	
C52	0.45417 (11)	0.48859 (9)	0.21120 (10)	0.0156 (4)	
H52	0.4961	0.5047	0.2001	0.019*	
C53	0.42506 (12)	0.42088 (10)	0.18881 (11)	0.0202 (4)	
H53	0.4472	0.3919	0.1630	0.024*	
C54	0.36389 (12)	0.39573 (10)	0.20406 (11)	0.0233 (4)	
H54	0.3433	0.3498	0.1881	0.028*	
C55	0.33328 (12)	0.43817 (10)	0.24267 (11)	0.0217 (4)	
H55	0.2921	0.4211	0.2544	0.026*	
C56	0.36271 (11)	0.50610 (10)	0.26462 (11)	0.0167 (4)	
H56	0.3404	0.5345	0.2907	0.020*	
C57	0.40442 (10)	0.66896 (9)	0.28797 (10)	0.0115 (3)	
C58	0.31882 (11)	0.67108 (9)	0.23425 (10)	0.0138 (3)	
H58	0.2938	0.6343	0.1958	0.017*	
C59	0.26885 (11)	0.72536 (10)	0.23514 (11)	0.0177 (4)	
H59	0.2112	0.7249	0.1978	0.021*	
C60	0.30362 (12)	0.77975 (10)	0.29049 (11)	0.0192 (4)	
H60	0.2701	0.8166	0.2916	0.023*	
C61	0.38795 (12)	0.77961 (10)	0.34415 (11)	0.0179 (4)	
H61	0.4126	0.8167	0.3822	0.021*	
C62	0.43671 (11)	0.72523 (9)	0.34240 (10)	0.0152 (4)	
H62	0.4944	0.7264	0.3798	0.018*	
C63	0.48849 (10)	0.64604 (9)	0.21412 (10)	0.0118 (3)	
C64	0.55235 (11)	0.69427 (9)	0.23561 (11)	0.0160 (4)	
H64	0.5889	0.7029	0.2924	0.019*	
C65	0.56471 (11)	0.73023 (10)	0.17738 (12)	0.0190 (4)	
H65	0.6084	0.7630	0.1948	0.023*	
C66	0.51356 (12)	0.71822 (10)	0.09466 (12)	0.0197 (4)	
H66	0.5219	0.7421	0.0546	0.024*	
C67	0.44959 (11)	0.67064 (10)	0.07073 (11)	0.0190 (4)	
H67	0.4138	0.6619	0.0138	0.023*	
C68	0.43761 (11)	0.63595 (10)	0.12913 (11)	0.0155 (3)	
H68	0.3931	0.6039	0.1110	0.019*	

### Atomic displacement parameters (Å^2^)

**Table:**

	*U*^11^	*U*^22^	*U*^33^	*U*^12^	*U*^13^	*U*^23^
N1	0.0144 (7)	0.0150 (7)	0.0125 (7)	0.0008 (6)	0.0063 (6)	0.0005 (6)
N2	0.0193 (8)	0.0144 (7)	0.0170 (7)	−0.0027 (6)	0.0116 (6)	−0.0031 (6)
N3	0.0141 (7)	0.0123 (7)	0.0149 (7)	0.0010 (6)	0.0078 (6)	0.0015 (6)
C1	0.0129 (8)	0.0149 (8)	0.0080 (7)	−0.0009 (7)	0.0028 (7)	−0.0005 (6)
C2	0.0196 (9)	0.0250 (10)	0.0174 (9)	0.0078 (8)	0.0096 (8)	0.0017 (7)
C3	0.0242 (10)	0.0123 (9)	0.0193 (9)	−0.0006 (7)	0.0086 (8)	−0.0030 (7)
C4	0.0281 (10)	0.0217 (10)	0.0213 (9)	−0.0056 (8)	0.0175 (8)	−0.0019 (8)
C5	0.0266 (10)	0.0138 (9)	0.0270 (10)	−0.0057 (7)	0.0171 (9)	−0.0070 (7)
C6	0.0286 (10)	0.0133 (9)	0.0267 (10)	0.0034 (8)	0.0164 (9)	0.0018 (7)
C7	0.0150 (9)	0.0123 (8)	0.0187 (9)	0.0025 (7)	0.0075 (7)	0.0006 (7)
C8	0.0170 (9)	0.0112 (8)	0.0165 (8)	−0.0011 (7)	0.0098 (7)	−0.0014 (6)
O1	0.0203 (7)	0.0209 (7)	0.0179 (6)	0.0054 (5)	0.0108 (6)	0.0053 (5)
O2	0.0187 (7)	0.0171 (7)	0.0243 (7)	0.0026 (5)	0.0144 (6)	0.0062 (5)
C9	0.0219 (10)	0.0184 (9)	0.0245 (9)	0.0015 (7)	0.0174 (8)	0.0048 (7)
C10	0.0275 (11)	0.0218 (10)	0.0284 (10)	−0.0031 (8)	0.0142 (9)	0.0038 (8)
N4	0.0144 (7)	0.0141 (7)	0.0140 (7)	−0.0013 (6)	0.0085 (6)	−0.0030 (6)
N5	0.0192 (8)	0.0233 (8)	0.0123 (7)	0.0010 (6)	0.0079 (6)	0.0004 (6)
N6	0.0123 (7)	0.0188 (8)	0.0133 (7)	0.0021 (6)	0.0056 (6)	0.0030 (6)
C11	0.0153 (8)	0.0084 (8)	0.0134 (8)	0.0020 (6)	0.0069 (7)	0.0023 (6)
C12	0.0159 (9)	0.0183 (9)	0.0173 (8)	−0.0027 (7)	0.0086 (7)	−0.0061 (7)
C13	0.0136 (9)	0.0192 (9)	0.0193 (9)	−0.0004 (7)	0.0097 (7)	−0.0023 (7)
C14	0.0363 (12)	0.0346 (12)	0.0204 (9)	0.0095 (9)	0.0204 (9)	0.0105 (9)
C15	0.0231 (10)	0.0330 (11)	0.0142 (9)	0.0029 (9)	0.0012 (8)	−0.0061 (8)
C16	0.0214 (10)	0.0223 (10)	0.0273 (10)	−0.0032 (8)	0.0033 (9)	−0.0020 (8)
C17	0.0122 (8)	0.0181 (9)	0.0179 (9)	0.0023 (7)	0.0043 (7)	0.0044 (7)
C18	0.0159 (9)	0.0136 (8)	0.0150 (8)	−0.0022 (7)	0.0087 (7)	−0.0022 (7)
O3	0.0179 (7)	0.0206 (7)	0.0178 (6)	0.0036 (5)	0.0104 (5)	0.0033 (5)
O4	0.0181 (6)	0.0189 (7)	0.0227 (7)	0.0026 (5)	0.0142 (6)	0.0041 (5)
C19	0.0222 (9)	0.0163 (9)	0.0205 (9)	−0.0009 (7)	0.0157 (8)	0.0009 (7)
C20	0.0295 (11)	0.0186 (10)	0.0253 (10)	−0.0027 (8)	0.0181 (9)	−0.0027 (8)
B1	0.0122 (9)	0.0110 (9)	0.0111 (8)	0.0009 (7)	0.0061 (7)	−0.0004 (7)
C21	0.0091 (8)	0.0154 (8)	0.0124 (8)	0.0028 (6)	0.0046 (7)	0.0006 (6)
C22	0.0146 (9)	0.0169 (9)	0.0153 (8)	−0.0017 (7)	0.0068 (7)	−0.0005 (7)
C23	0.0186 (9)	0.0206 (9)	0.0196 (9)	−0.0018 (7)	0.0099 (8)	0.0042 (7)
C24	0.0164 (9)	0.0268 (10)	0.0111 (8)	0.0058 (7)	0.0080 (7)	0.0035 (7)
C25	0.0168 (9)	0.0213 (9)	0.0113 (8)	0.0027 (7)	0.0039 (7)	−0.0031 (7)
C26	0.0155 (9)	0.0123 (8)	0.0154 (8)	−0.0002 (7)	0.0072 (7)	−0.0006 (7)
C27	0.0142 (8)	0.0081 (8)	0.0124 (8)	0.0022 (6)	0.0052 (7)	0.0007 (6)
C28	0.0156 (9)	0.0146 (8)	0.0135 (8)	0.0005 (7)	0.0068 (7)	−0.0008 (6)
C29	0.0119 (8)	0.0159 (9)	0.0208 (9)	0.0005 (7)	0.0063 (7)	−0.0005 (7)
C30	0.0150 (9)	0.0117 (8)	0.0148 (8)	0.0026 (7)	0.0016 (7)	−0.0003 (6)
C31	0.0241 (10)	0.0131 (8)	0.0116 (8)	0.0043 (7)	0.0070 (7)	0.0012 (6)
C32	0.0155 (8)	0.0133 (8)	0.0163 (8)	0.0013 (7)	0.0081 (7)	0.0006 (7)
C33	0.0140 (8)	0.0124 (8)	0.0105 (7)	0.0006 (6)	0.0079 (7)	0.0020 (6)
C34	0.0151 (8)	0.0149 (8)	0.0122 (8)	−0.0018 (7)	0.0067 (7)	−0.0015 (6)
C35	0.0123 (8)	0.0248 (10)	0.0142 (8)	0.0006 (7)	0.0070 (7)	0.0028 (7)
C36	0.0189 (9)	0.0195 (9)	0.0206 (9)	0.0096 (7)	0.0121 (8)	0.0072 (7)
C37	0.0272 (10)	0.0112 (9)	0.0202 (9)	0.0034 (7)	0.0109 (8)	0.0007 (7)
C38	0.0135 (8)	0.0150 (9)	0.0161 (8)	−0.0008 (7)	0.0054 (7)	−0.0014 (7)
C39	0.0150 (8)	0.0135 (8)	0.0078 (7)	0.0005 (7)	0.0045 (7)	−0.0004 (6)
C40	0.0179 (9)	0.0182 (9)	0.0151 (8)	0.0019 (7)	0.0086 (7)	0.0002 (7)
C41	0.0260 (10)	0.0153 (9)	0.0141 (8)	0.0078 (7)	0.0068 (8)	0.0013 (7)
C42	0.0344 (11)	0.0129 (9)	0.0138 (8)	−0.0025 (8)	0.0080 (8)	0.0012 (7)
C43	0.0255 (10)	0.0172 (9)	0.0179 (9)	−0.0056 (8)	0.0124 (8)	0.0008 (7)
C44	0.0202 (9)	0.0140 (8)	0.0155 (8)	−0.0003 (7)	0.0098 (7)	−0.0015 (7)
B2	0.0113 (9)	0.0102 (9)	0.0098 (8)	−0.0013 (7)	0.0055 (7)	−0.0015 (7)
C45	0.0134 (8)	0.0090 (8)	0.0130 (8)	−0.0008 (6)	0.0042 (7)	−0.0030 (6)
C46	0.0160 (9)	0.0118 (8)	0.0208 (9)	−0.0004 (7)	0.0067 (8)	−0.0028 (7)
C47	0.0126 (9)	0.0141 (9)	0.0281 (10)	0.0017 (7)	0.0012 (8)	−0.0037 (8)
C48	0.0239 (10)	0.0123 (9)	0.0179 (9)	0.0014 (7)	−0.0045 (8)	−0.0032 (7)
C49	0.0310 (11)	0.0184 (10)	0.0122 (8)	0.0024 (8)	0.0059 (8)	−0.0010 (7)
C50	0.0181 (9)	0.0166 (9)	0.0155 (8)	0.0016 (7)	0.0066 (7)	−0.0011 (7)
C51	0.0107 (8)	0.0134 (8)	0.0066 (7)	−0.0004 (6)	0.0006 (6)	0.0006 (6)
C52	0.0136 (8)	0.0160 (9)	0.0136 (8)	0.0026 (7)	0.0038 (7)	0.0005 (7)
C53	0.0209 (10)	0.0141 (9)	0.0166 (9)	0.0030 (7)	0.0022 (8)	−0.0031 (7)
C54	0.0232 (10)	0.0122 (9)	0.0192 (9)	−0.0028 (7)	−0.0015 (8)	0.0007 (7)
C55	0.0180 (9)	0.0203 (10)	0.0190 (9)	−0.0067 (8)	0.0029 (8)	0.0052 (7)
C56	0.0172 (9)	0.0176 (9)	0.0139 (8)	−0.0017 (7)	0.0065 (7)	0.0020 (7)
C57	0.0139 (8)	0.0123 (8)	0.0109 (7)	0.0000 (6)	0.0082 (7)	0.0022 (6)
C58	0.0163 (9)	0.0152 (8)	0.0122 (8)	0.0003 (7)	0.0087 (7)	0.0027 (6)
C59	0.0156 (9)	0.0239 (10)	0.0161 (8)	0.0062 (7)	0.0096 (7)	0.0098 (7)
C60	0.0273 (10)	0.0191 (9)	0.0203 (9)	0.0106 (8)	0.0186 (8)	0.0086 (7)
C61	0.0293 (10)	0.0138 (9)	0.0152 (8)	0.0018 (7)	0.0145 (8)	0.0003 (7)
C62	0.0166 (9)	0.0160 (9)	0.0130 (8)	0.0004 (7)	0.0073 (7)	0.0011 (7)
C63	0.0105 (8)	0.0113 (8)	0.0151 (8)	0.0030 (6)	0.0075 (7)	0.0000 (6)
C64	0.0148 (9)	0.0157 (9)	0.0180 (9)	0.0001 (7)	0.0086 (7)	−0.0022 (7)
C65	0.0167 (9)	0.0156 (9)	0.0303 (10)	−0.0014 (7)	0.0159 (8)	0.0002 (8)
C66	0.0233 (10)	0.0187 (9)	0.0267 (10)	0.0068 (8)	0.0196 (8)	0.0064 (8)
C67	0.0186 (9)	0.0236 (10)	0.0151 (8)	0.0047 (7)	0.0086 (8)	0.0022 (7)
C68	0.0132 (8)	0.0176 (9)	0.0154 (8)	−0.0008 (7)	0.0069 (7)	−0.0005 (7)

### Geometric parameters (Å, º)

**Table:**

N1—C1	1.323 (2)	C25—H25	0.9500
N1—C3	1.460 (2)	C26—H26	0.9500
N1—C2	1.465 (2)	C27—C28	1.400 (2)
N2—C1	1.333 (2)	C27—C32	1.404 (2)
N2—C4	1.464 (2)	C28—C29	1.398 (2)
N2—C5	1.477 (2)	C28—H28	0.9500
N3—C1	1.379 (2)	C29—C30	1.382 (3)
N3—C8	1.406 (2)	C29—H29	0.9500
N3—C7	1.495 (2)	C30—C31	1.391 (3)
C2—H2A	0.9800	C30—H30	0.9500
C2—H2B	0.9800	C31—C32	1.388 (2)
C2—H2C	0.9800	C31—H31	0.9500
C3—H3A	0.9800	C32—H32	0.9500
C3—H3B	0.9800	C33—C38	1.400 (2)
C3—H3C	0.9800	C33—C34	1.408 (2)
C4—H4A	0.9800	C34—C35	1.387 (3)
C4—H4B	0.9800	C34—H34	0.9500
C4—H4C	0.9800	C35—C36	1.391 (3)
C5—C6	1.511 (3)	C35—H35	0.9500
C5—H5A	0.9900	C36—C37	1.381 (3)
C5—H5B	0.9900	C36—H36	0.9500
C6—C7	1.521 (3)	C37—C38	1.391 (3)
C6—H6A	0.9900	C37—H37	0.9500
C6—H6B	0.9900	C38—H38	0.9500
C7—H7A	0.9900	C39—C44	1.400 (2)
C7—H7B	0.9900	C39—C40	1.411 (2)
C8—O1	1.199 (2)	C40—C41	1.386 (3)
C8—O2	1.331 (2)	C40—H40	0.9500
O2—C9	1.465 (2)	C41—C42	1.386 (3)
C9—C10	1.499 (3)	C41—H41	0.9500
C9—H9A	0.9900	C42—C43	1.380 (3)
C9—H9B	0.9900	C42—H42	0.9500
C10—H10A	0.9800	C43—C44	1.405 (3)
C10—H10B	0.9800	C43—H43	0.9500
C10—H10C	0.9800	C44—H44	0.9500
N4—C11	1.326 (2)	B2—C51	1.638 (2)
N4—C12	1.464 (2)	B2—C63	1.643 (2)
N4—C13	1.472 (2)	B2—C45	1.643 (2)
N5—C11	1.324 (2)	B2—C57	1.645 (2)
N5—C14	1.463 (2)	C45—C46	1.405 (2)
N5—C15	1.474 (2)	C45—C50	1.405 (2)
N6—C11	1.381 (2)	C46—C47	1.391 (3)
N6—C18	1.409 (2)	C46—H46	0.9500
N6—C17	1.482 (2)	C47—C48	1.384 (3)
C12—H12A	0.9800	C47—H47	0.9500
C12—H12B	0.9800	C48—C49	1.383 (3)
C12—H12C	0.9800	C48—H48	0.9500
C13—H13A	0.9800	C49—C50	1.392 (3)
C13—H13B	0.9800	C49—H49	0.9500
C13—H13C	0.9800	C50—H50	0.9500
C14—H14A	0.9800	C51—C52	1.404 (2)
C14—H14B	0.9800	C51—C56	1.407 (2)
C14—H14C	0.9800	C52—C53	1.392 (3)
C15—C16	1.506 (3)	C52—H52	0.9500
C15—H15A	0.9900	C53—C54	1.386 (3)
C15—H15B	0.9900	C53—H53	0.9500
C16—C17	1.501 (3)	C54—C55	1.380 (3)
C16—H16A	0.9900	C54—H54	0.9500
C16—H16B	0.9900	C55—C56	1.397 (3)
C17—H17A	0.9900	C55—H55	0.9500
C17—H17B	0.9900	C56—H56	0.9500
C18—O3	1.202 (2)	C57—C62	1.400 (2)
C18—O4	1.329 (2)	C57—C58	1.405 (2)
O4—C19	1.456 (2)	C58—C59	1.402 (3)
C19—C20	1.504 (3)	C58—H58	0.9500
C19—H19A	0.9900	C59—C60	1.386 (3)
C19—H19B	0.9900	C59—H59	0.9500
C20—H20A	0.9800	C60—C61	1.386 (3)
C20—H20B	0.9800	C60—H60	0.9500
C20—H20C	0.9800	C61—C62	1.393 (3)
B1—C27	1.640 (2)	C61—H61	0.9500
B1—C33	1.640 (2)	C62—H62	0.9500
B1—C21	1.644 (2)	C63—C64	1.401 (2)
B1—C39	1.645 (3)	C63—C68	1.404 (2)
C21—C26	1.399 (2)	C64—C65	1.396 (3)
C21—C22	1.408 (2)	C64—H64	0.9500
C22—C23	1.387 (3)	C65—C66	1.377 (3)
C22—H22	0.9500	C65—H65	0.9500
C23—C24	1.384 (3)	C66—C67	1.389 (3)
C23—H23	0.9500	C66—H66	0.9500
C24—C25	1.385 (3)	C67—C68	1.383 (3)
C24—H24	0.9500	C67—H67	0.9500
C25—C26	1.390 (2)	C68—H68	0.9500
			
C1—N1—C3	123.43 (15)	C22—C23—H23	120.0
C1—N1—C2	122.60 (15)	C23—C24—C25	119.17 (16)
C3—N1—C2	113.85 (15)	C23—C24—H24	120.4
C1—N2—C4	122.45 (15)	C25—C24—H24	120.4
C1—N2—C5	115.92 (14)	C24—C25—C26	120.10 (17)
C4—N2—C5	117.00 (15)	C24—C25—H25	119.9
C1—N3—C8	120.35 (14)	C26—C25—H25	119.9
C1—N3—C7	120.94 (14)	C25—C26—C21	122.79 (17)
C8—N3—C7	118.37 (14)	C25—C26—H26	118.6
N1—C1—N2	122.69 (16)	C21—C26—H26	118.6
N1—C1—N3	120.46 (15)	C28—C27—C32	115.13 (15)
N2—C1—N3	116.84 (15)	C28—C27—B1	126.56 (15)
N1—C2—H2A	109.5	C32—C27—B1	118.29 (15)
N1—C2—H2B	109.5	C29—C28—C27	122.91 (16)
H2A—C2—H2B	109.5	C29—C28—H28	118.5
N1—C2—H2C	109.5	C27—C28—H28	118.5
H2A—C2—H2C	109.5	C30—C29—C28	119.99 (17)
H2B—C2—H2C	109.5	C30—C29—H29	120.0
N1—C3—H3A	109.5	C28—C29—H29	120.0
N1—C3—H3B	109.5	C29—C30—C31	118.90 (16)
H3A—C3—H3B	109.5	C29—C30—H30	120.5
N1—C3—H3C	109.5	C31—C30—H30	120.5
H3A—C3—H3C	109.5	C32—C31—C30	120.24 (16)
H3B—C3—H3C	109.5	C32—C31—H31	119.9
N2—C4—H4A	109.5	C30—C31—H31	119.9
N2—C4—H4B	109.5	C31—C32—C27	122.83 (17)
H4A—C4—H4B	109.5	C31—C32—H32	118.6
N2—C4—H4C	109.5	C27—C32—H32	118.6
H4A—C4—H4C	109.5	C38—C33—C34	115.37 (15)
H4B—C4—H4C	109.5	C38—C33—B1	124.00 (15)
N2—C5—C6	106.66 (15)	C34—C33—B1	120.32 (15)
N2—C5—H5A	110.4	C35—C34—C33	122.77 (16)
C6—C5—H5A	110.4	C35—C34—H34	118.6
N2—C5—H5B	110.4	C33—C34—H34	118.6
C6—C5—H5B	110.4	C34—C35—C36	120.13 (17)
H5A—C5—H5B	108.6	C34—C35—H35	119.9
C5—C6—C7	108.33 (15)	C36—C35—H35	119.9
C5—C6—H6A	110.0	C37—C36—C35	118.53 (17)
C7—C6—H6A	110.0	C37—C36—H36	120.7
C5—C6—H6B	110.0	C35—C36—H36	120.7
C7—C6—H6B	110.0	C36—C37—C38	120.91 (17)
H6A—C6—H6B	108.4	C36—C37—H37	119.5
N3—C7—C6	111.26 (15)	C38—C37—H37	119.5
N3—C7—H7A	109.4	C37—C38—C33	122.27 (17)
C6—C7—H7A	109.4	C37—C38—H38	118.9
N3—C7—H7B	109.4	C33—C38—H38	118.9
C6—C7—H7B	109.4	C44—C39—C40	115.27 (16)
H7A—C7—H7B	108.0	C44—C39—B1	125.15 (15)
O1—C8—O2	126.89 (16)	C40—C39—B1	119.51 (15)
O1—C8—N3	124.10 (16)	C41—C40—C39	122.80 (17)
O2—C8—N3	109.02 (14)	C41—C40—H40	118.6
C8—O2—C9	115.89 (14)	C39—C40—H40	118.6
O2—C9—C10	110.73 (15)	C42—C41—C40	120.11 (18)
O2—C9—H9A	109.5	C42—C41—H41	119.9
C10—C9—H9A	109.5	C40—C41—H41	119.9
O2—C9—H9B	109.5	C43—C42—C41	119.36 (18)
C10—C9—H9B	109.5	C43—C42—H42	120.3
H9A—C9—H9B	108.1	C41—C42—H42	120.3
C9—C10—H10A	109.5	C42—C43—C44	119.98 (18)
C9—C10—H10B	109.5	C42—C43—H43	120.0
H10A—C10—H10B	109.5	C44—C43—H43	120.0
C9—C10—H10C	109.5	C39—C44—C43	122.44 (17)
H10A—C10—H10C	109.5	C39—C44—H44	118.8
H10B—C10—H10C	109.5	C43—C44—H44	118.8
C11—N4—C12	121.76 (15)	C51—B2—C63	110.53 (13)
C11—N4—C13	122.96 (14)	C51—B2—C45	104.41 (13)
C12—N4—C13	114.62 (14)	C63—B2—C45	113.29 (14)
C11—N5—C14	121.88 (16)	C51—B2—C57	114.51 (14)
C11—N5—C15	123.72 (16)	C63—B2—C57	102.97 (13)
C14—N5—C15	114.09 (15)	C45—B2—C57	111.43 (13)
C11—N6—C18	118.74 (14)	C46—C45—C50	115.44 (16)
C11—N6—C17	116.00 (14)	C46—C45—B2	123.48 (15)
C18—N6—C17	118.83 (14)	C50—C45—B2	120.84 (15)
N5—C11—N4	122.18 (16)	C47—C46—C45	122.56 (18)
N5—C11—N6	117.57 (15)	C47—C46—H46	118.7
N4—C11—N6	120.11 (15)	C45—C46—H46	118.7
N4—C12—H12A	109.5	C48—C47—C46	120.02 (18)
N4—C12—H12B	109.5	C48—C47—H47	120.0
H12A—C12—H12B	109.5	C46—C47—H47	120.0
N4—C12—H12C	109.5	C49—C48—C47	119.43 (17)
H12A—C12—H12C	109.5	C49—C48—H48	120.3
H12B—C12—H12C	109.5	C47—C48—H48	120.3
N4—C13—H13A	109.5	C48—C49—C50	119.97 (18)
N4—C13—H13B	109.5	C48—C49—H49	120.0
H13A—C13—H13B	109.5	C50—C49—H49	120.0
N4—C13—H13C	109.5	C49—C50—C45	122.54 (18)
H13A—C13—H13C	109.5	C49—C50—H50	118.7
H13B—C13—H13C	109.5	C45—C50—H50	118.7
N5—C14—H14A	109.5	C52—C51—C56	115.31 (16)
N5—C14—H14B	109.5	C52—C51—B2	120.42 (15)
H14A—C14—H14B	109.5	C56—C51—B2	124.02 (15)
N5—C14—H14C	109.5	C53—C52—C51	122.68 (17)
H14A—C14—H14C	109.5	C53—C52—H52	118.7
H14B—C14—H14C	109.5	C51—C52—H52	118.7
N5—C15—C16	113.56 (16)	C54—C53—C52	120.12 (18)
N5—C15—H15A	108.9	C54—C53—H53	119.9
C16—C15—H15A	108.9	C52—C53—H53	119.9
N5—C15—H15B	108.9	C55—C54—C53	119.23 (17)
C16—C15—H15B	108.9	C55—C54—H54	120.4
H15A—C15—H15B	107.7	C53—C54—H54	120.4
C17—C16—C15	108.01 (17)	C54—C55—C56	120.19 (18)
C17—C16—H16A	110.1	C54—C55—H55	119.9
C15—C16—H16A	110.1	C56—C55—H55	119.9
C17—C16—H16B	110.1	C55—C56—C51	122.46 (18)
C15—C16—H16B	110.1	C55—C56—H56	118.8
H16A—C16—H16B	108.4	C51—C56—H56	118.8
N6—C17—C16	105.98 (15)	C62—C57—C58	115.31 (16)
N6—C17—H17A	110.5	C62—C57—B2	120.82 (15)
C16—C17—H17A	110.5	C58—C57—B2	123.41 (15)
N6—C17—H17B	110.5	C59—C58—C57	122.58 (17)
C16—C17—H17B	110.5	C59—C58—H58	118.7
H17A—C17—H17B	108.7	C57—C58—H58	118.7
O3—C18—O4	126.37 (16)	C60—C59—C58	119.97 (17)
O3—C18—N6	124.48 (16)	C60—C59—H59	120.0
O4—C18—N6	109.15 (15)	C58—C59—H59	120.0
C18—O4—C19	115.60 (14)	C59—C60—C61	119.07 (17)
O4—C19—C20	110.62 (15)	C59—C60—H60	120.5
O4—C19—H19A	109.5	C61—C60—H60	120.5
C20—C19—H19A	109.5	C60—C61—C62	120.19 (17)
O4—C19—H19B	109.5	C60—C61—H61	119.9
C20—C19—H19B	109.5	C62—C61—H61	119.9
H19A—C19—H19B	108.1	C61—C62—C57	122.88 (17)
C19—C20—H20A	109.5	C61—C62—H62	118.6
C19—C20—H20B	109.5	C57—C62—H62	118.6
H20A—C20—H20B	109.5	C64—C63—C68	114.99 (16)
C19—C20—H20C	109.5	C64—C63—B2	123.41 (15)
H20A—C20—H20C	109.5	C68—C63—B2	121.14 (15)
H20B—C20—H20C	109.5	C65—C64—C63	122.94 (17)
C27—B1—C33	110.48 (14)	C65—C64—H64	118.5
C27—B1—C21	114.04 (14)	C63—C64—H64	118.5
C33—B1—C21	102.80 (13)	C66—C65—C64	119.99 (17)
C27—B1—C39	106.22 (13)	C66—C65—H65	120.0
C33—B1—C39	113.27 (14)	C64—C65—H65	120.0
C21—B1—C39	110.23 (14)	C65—C66—C67	118.89 (17)
C26—C21—C22	115.13 (15)	C65—C66—H66	120.6
C26—C21—B1	122.76 (15)	C67—C66—H66	120.6
C22—C21—B1	121.83 (15)	C68—C67—C66	120.45 (17)
C23—C22—C21	122.84 (17)	C68—C67—H67	119.8
C23—C22—H22	118.6	C66—C67—H67	119.8
C21—C22—H22	118.6	C67—C68—C63	122.73 (17)
C24—C23—C22	119.94 (17)	C67—C68—H68	118.6
C24—C23—H23	120.0	C63—C68—H68	118.6
			
C3—N1—C1—N2	−157.48 (16)	C27—B1—C33—C34	−168.69 (15)
C2—N1—C1—N2	26.7 (2)	C21—B1—C33—C34	69.26 (18)
C3—N1—C1—N3	21.4 (2)	C39—B1—C33—C34	−49.7 (2)
C2—N1—C1—N3	−154.40 (15)	C38—C33—C34—C35	−1.0 (2)
C4—N2—C1—N1	33.4 (3)	B1—C33—C34—C35	−174.89 (16)
C5—N2—C1—N1	−171.44 (16)	C33—C34—C35—C36	1.0 (3)
C4—N2—C1—N3	−145.49 (17)	C34—C35—C36—C37	0.1 (3)
C5—N2—C1—N3	9.6 (2)	C35—C36—C37—C38	−1.2 (3)
C8—N3—C1—N1	41.4 (2)	C36—C37—C38—C33	1.1 (3)
C7—N3—C1—N1	−145.28 (16)	C34—C33—C38—C37	−0.1 (2)
C8—N3—C1—N2	−139.61 (16)	B1—C33—C38—C37	173.56 (16)
C7—N3—C1—N2	33.7 (2)	C27—B1—C39—C44	110.49 (18)
C1—N2—C5—C6	−58.4 (2)	C33—B1—C39—C44	−11.0 (2)
C4—N2—C5—C6	98.17 (18)	C21—B1—C39—C44	−125.50 (17)
N2—C5—C6—C7	63.43 (19)	C27—B1—C39—C40	−66.51 (18)
C1—N3—C7—C6	−23.6 (2)	C33—B1—C39—C40	172.05 (14)
C8—N3—C7—C6	149.82 (16)	C21—B1—C39—C40	57.5 (2)
C5—C6—C7—N3	−25.2 (2)	C44—C39—C40—C41	2.2 (2)
C1—N3—C8—O1	16.0 (3)	B1—C39—C40—C41	179.49 (16)
C7—N3—C8—O1	−157.45 (17)	C39—C40—C41—C42	−1.1 (3)
C1—N3—C8—O2	−164.46 (15)	C40—C41—C42—C43	−0.4 (3)
C7—N3—C8—O2	22.1 (2)	C41—C42—C43—C44	0.8 (3)
O1—C8—O2—C9	3.0 (3)	C40—C39—C44—C43	−1.8 (2)
N3—C8—O2—C9	−176.55 (14)	B1—C39—C44—C43	−178.92 (16)
C8—O2—C9—C10	−84.87 (19)	C42—C43—C44—C39	0.4 (3)
C14—N5—C11—N4	19.6 (3)	C51—B2—C45—C46	88.03 (19)
C15—N5—C11—N4	−167.15 (17)	C63—B2—C45—C46	−32.3 (2)
C14—N5—C11—N6	−155.88 (17)	C57—B2—C45—C46	−147.84 (16)
C15—N5—C11—N6	17.3 (3)	C51—B2—C45—C50	−86.17 (18)
C12—N4—C11—N5	−152.35 (17)	C63—B2—C45—C50	153.51 (16)
C13—N4—C11—N5	37.4 (3)	C57—B2—C45—C50	38.0 (2)
C12—N4—C11—N6	23.1 (2)	C50—C45—C46—C47	−1.3 (3)
C13—N4—C11—N6	−147.19 (16)	B2—C45—C46—C47	−175.75 (16)
C18—N6—C11—N5	−133.56 (17)	C45—C46—C47—C48	0.4 (3)
C17—N6—C11—N5	18.0 (2)	C46—C47—C48—C49	0.7 (3)
C18—N6—C11—N4	50.8 (2)	C47—C48—C49—C50	−1.0 (3)
C17—N6—C11—N4	−157.63 (16)	C48—C49—C50—C45	0.1 (3)
C11—N5—C15—C16	−8.6 (3)	C46—C45—C50—C49	1.0 (3)
C14—N5—C15—C16	165.11 (18)	B2—C45—C50—C49	175.67 (17)
N5—C15—C16—C17	−32.7 (2)	C63—B2—C51—C52	39.9 (2)
C11—N6—C17—C16	−58.6 (2)	C45—B2—C51—C52	−82.29 (18)
C18—N6—C17—C16	92.93 (19)	C57—B2—C51—C52	155.59 (15)
C15—C16—C17—N6	63.05 (19)	C63—B2—C51—C56	−146.22 (16)
C11—N6—C18—O3	−3.9 (3)	C45—B2—C51—C56	91.62 (18)
C17—N6—C18—O3	−154.68 (17)	C57—B2—C51—C56	−30.5 (2)
C11—N6—C18—O4	176.03 (15)	C56—C51—C52—C53	0.6 (2)
C17—N6—C18—O4	25.3 (2)	B2—C51—C52—C53	175.06 (15)
O3—C18—O4—C19	7.9 (3)	C51—C52—C53—C54	0.0 (3)
N6—C18—O4—C19	−172.08 (14)	C52—C53—C54—C55	−1.0 (3)
C18—O4—C19—C20	80.33 (19)	C53—C54—C55—C56	1.2 (3)
C27—B1—C21—C26	137.68 (16)	C54—C55—C56—C51	−0.5 (3)
C33—B1—C21—C26	−102.71 (18)	C52—C51—C56—C55	−0.4 (2)
C39—B1—C21—C26	18.3 (2)	B2—C51—C56—C55	−174.60 (16)
C27—B1—C21—C22	−48.7 (2)	C51—B2—C57—C62	154.21 (15)
C33—B1—C21—C22	70.94 (19)	C63—B2—C57—C62	−85.76 (17)
C39—B1—C21—C22	−168.04 (15)	C45—B2—C57—C62	36.0 (2)
C26—C21—C22—C23	−2.1 (3)	C51—B2—C57—C58	−33.9 (2)
B1—C21—C22—C23	−176.23 (16)	C63—B2—C57—C58	86.11 (18)
C21—C22—C23—C24	1.5 (3)	C45—B2—C57—C58	−152.13 (15)
C22—C23—C24—C25	0.4 (3)	C62—C57—C58—C59	−0.4 (2)
C23—C24—C25—C26	−1.4 (3)	B2—C57—C58—C59	−172.67 (15)
C24—C25—C26—C21	0.6 (3)	C57—C58—C59—C60	0.0 (3)
C22—C21—C26—C25	1.1 (3)	C58—C59—C60—C61	0.3 (3)
B1—C21—C26—C25	175.13 (16)	C59—C60—C61—C62	−0.3 (3)
C33—B1—C27—C28	−121.25 (18)	C60—C61—C62—C57	−0.1 (3)
C21—B1—C27—C28	−6.1 (2)	C58—C57—C62—C61	0.4 (2)
C39—B1—C27—C28	115.53 (18)	B2—C57—C62—C61	172.91 (16)
C33—B1—C27—C32	60.7 (2)	C51—B2—C63—C64	−152.35 (16)
C21—B1—C27—C32	175.83 (15)	C45—B2—C63—C64	−35.6 (2)
C39—B1—C27—C32	−62.57 (19)	C57—B2—C63—C64	84.91 (19)
C32—C27—C28—C29	−0.3 (3)	C51—B2—C63—C68	35.8 (2)
B1—C27—C28—C29	−178.48 (16)	C45—B2—C63—C68	152.59 (15)
C27—C28—C29—C30	0.1 (3)	C57—B2—C63—C68	−86.92 (18)
C28—C29—C30—C31	0.4 (3)	C68—C63—C64—C65	0.4 (3)
C29—C30—C31—C32	−0.5 (3)	B2—C63—C64—C65	−171.90 (16)
C30—C31—C32—C27	0.2 (3)	C63—C64—C65—C66	−0.9 (3)
C28—C27—C32—C31	0.2 (3)	C64—C65—C66—C67	0.7 (3)
B1—C27—C32—C31	178.48 (16)	C65—C66—C67—C68	0.0 (3)
C27—B1—C33—C38	18.0 (2)	C66—C67—C68—C63	−0.5 (3)
C21—B1—C33—C38	−104.06 (18)	C64—C63—C68—C67	0.3 (3)
C39—B1—C33—C38	137.01 (16)	B2—C63—C68—C67	172.78 (16)

### Hydrogen-bond geometry (Å, º)

Cg1, Cg2, Cg3, Cg4, Cg5 and Cg6 are the centroids of the C21–C26, C27–C32, C45–C50, C51–C56, C57–C62 and C63–C68 rings, respectively.

**Table:**

*D*—H···*A*	*D*—H	H···*A*	*D*···*A*	*D*—H···*A*
C5—H5*A*···*Cg*1	0.99	2.77	3.502 (3)	131
C19—H19*B*···*Cg*1	0.99	2.86	3.559 (3)	128
C17—H17*B*···*Cg*2	0.99	2.67	3.632 (3)	165
C6—H6*B*···*Cg*3	0.99	2.75	3.624 (3)	147
C13—H13*C*···*Cg*3	0.98	2.72	3.421 (3)	129
C13—H13*B*···*Cg*4	0.98	2.67	3.583 (3)	154
C12—H12*C*···*Cg*5	0.98	2.70	3.329 (3)	121
C14—H14*B*···*Cg*5	0.98	2.76	3.354 (3)	120
C12—H12*B*···*Cg*6	0.98	2.64	3.535 (3)	151
C9—H9*A*···*Cg*6	0.99	2.75	3.655 (3)	151
