# Supplementary figures and images for: Crystal structure of 2-di­methyl­amino-1-eth­oxy­carbonyl-3-methyl-3,4,5,6-tetra­hydro­pyrimidin-1-ium tetra­phenyl­borate
Source: Acta Crystallogr E Crystallogr Commun. 2015 Oct 31;71(Pt 11):o894–5. doi: 10.1107/S2056989015020034 (PMC4645042; doi:10.1107/S2056989015020034)

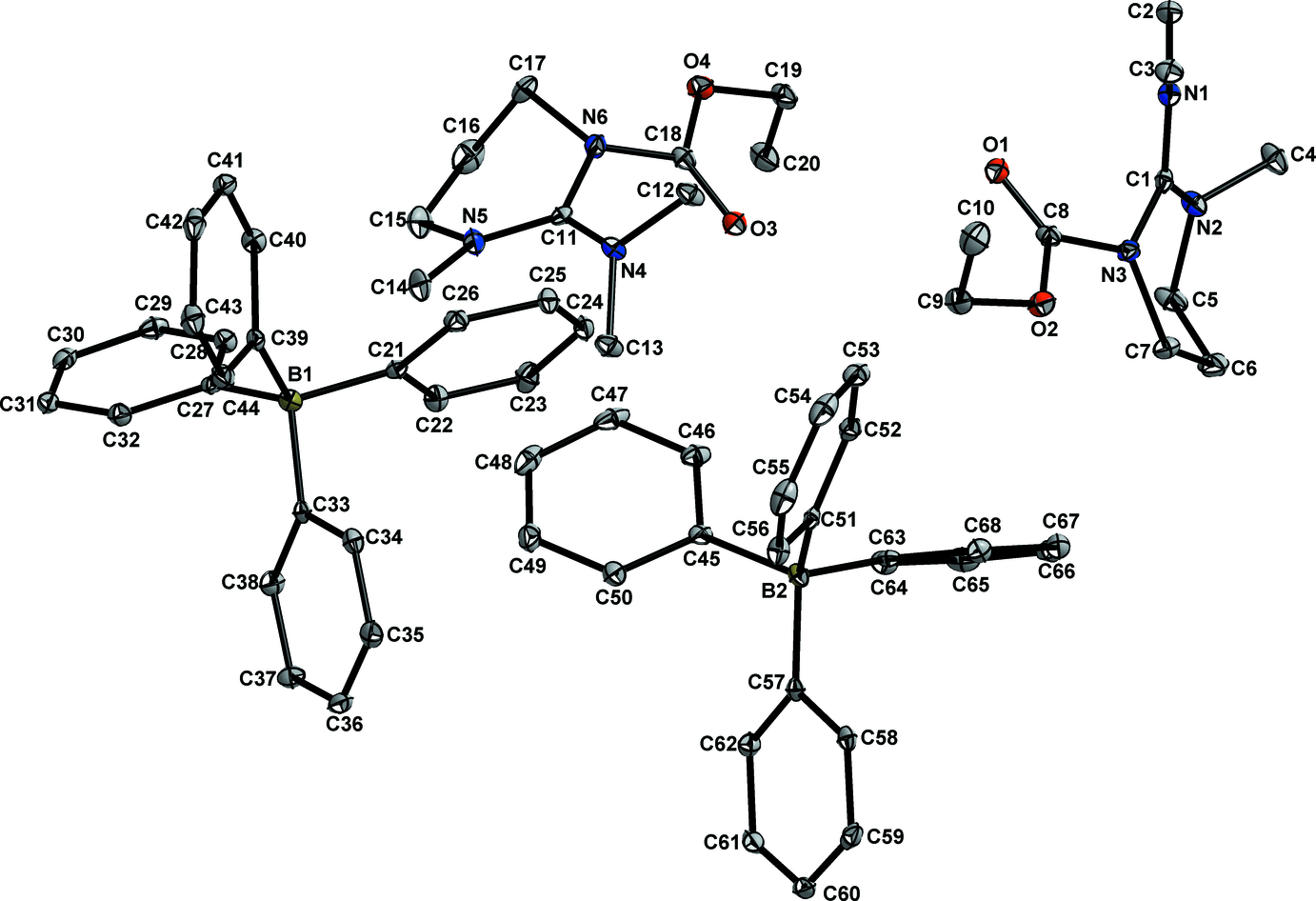

Supplement: Supplementary file 4 [file e-71-0o894-fig1.tif]

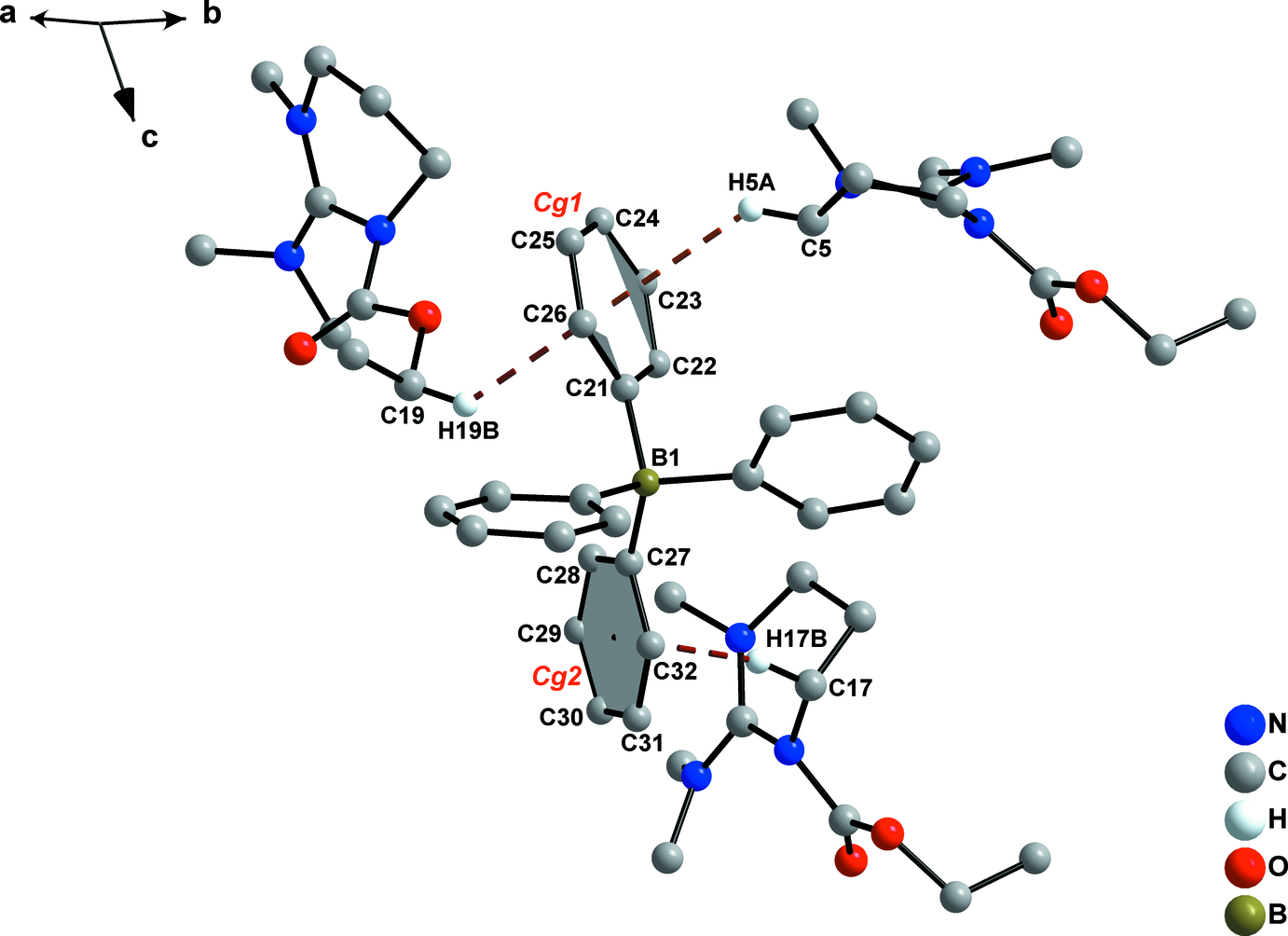

Supplement: Supplementary file 5 [file e-71-0o894-fig2.tif]

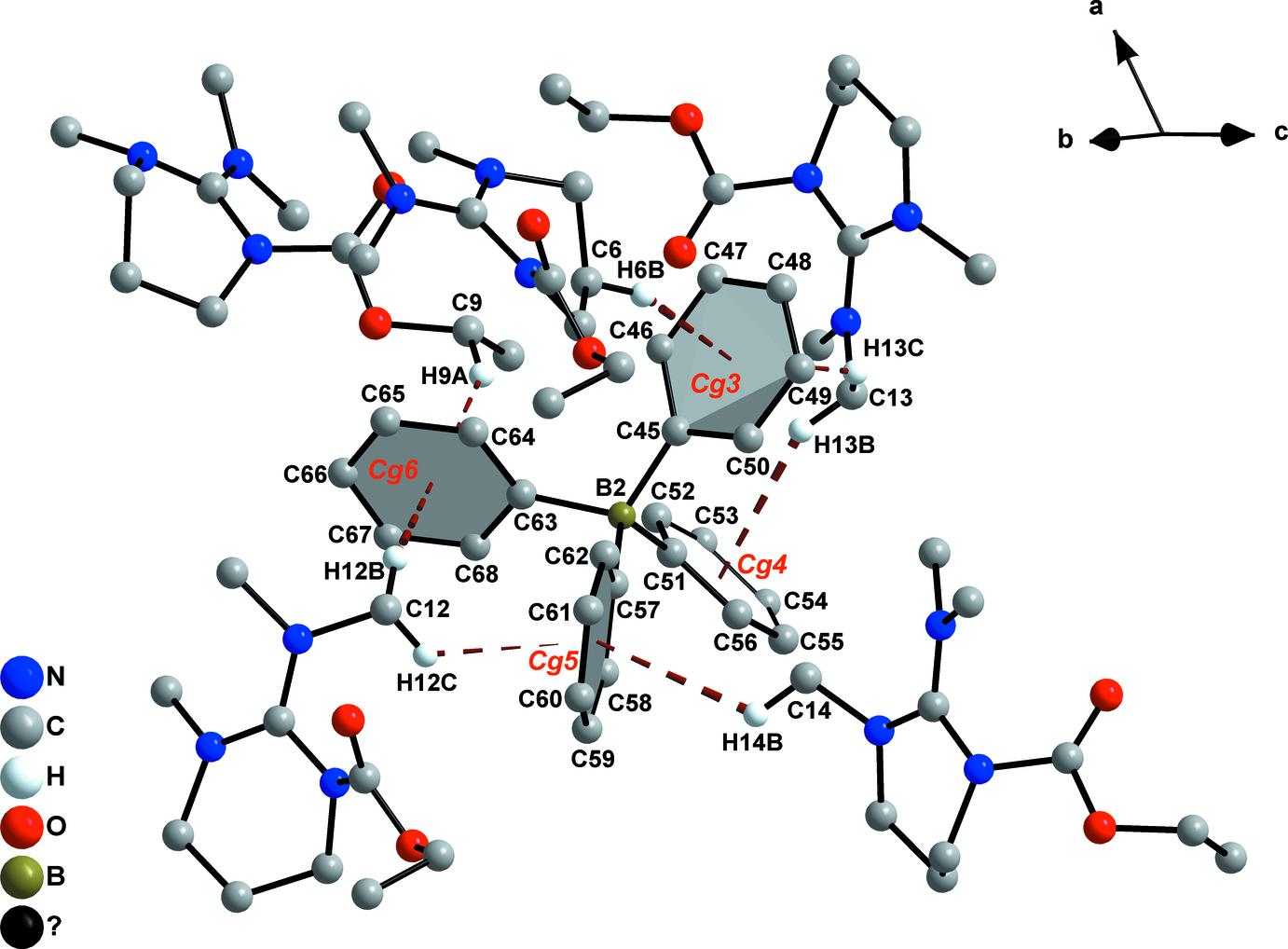

Supplement: Supplementary file 6 [file e-71-0o894-fig3.tif]
